# Dermoscopy of lipidized dermatofibromas^⋆^

**DOI:** 10.1016/j.abd.2021.12.010

**Published:** 2023-02-17

**Authors:** Tugba Kevser Uzuncakmak, Muazzez Cigdem Oba, Mehmet Sar, Zekayi Kutlubay

**Affiliations:** a Department of Dermatology and Venereology, Istanbul University-Cerrahpasa, Cerrahpaşa Medical Faculty, Istanbul, Turkey; b Department of Pathology, Istanbul University-Cerrahpasa, Cerrahpasa Medical Faculty, Istanbul, Turkey

Dear Editor,

A 17-year-old boy without medical antecedents presented to the dermatology clinic with a six-month history of papular lesions on his lower extremities. Dermatologic examination revealed three asymptomatic, firm, yellow-brown papules, scattered on the lower extremities (Fig. 1A–C). On dermoscopy, a yellow homogenous area with the central white network, surrounded by a pinkish halo, was seen (Fig. 2A–C). Routine blood tests showed normal full blood count, renal and liver biochemistry. A punch biopsy of a papule was performed for light microscopy (Fig. 3A–C). Low power view revealed a hypercellular lesion with increased collagen fibers, extending from the superficial dermis to the deep dermis (Fig. 3A). Abundant foamy histiocytes were seen among dense collagen fibers (Fig. 3B–C). Immunohistochemically, these cells with vacuolar cytoplasm were CD68 positive (Fig. 3D). Given the clinicopathologic findings, lipidized dermatofibroma was diagnosed.

**Figure 1:**
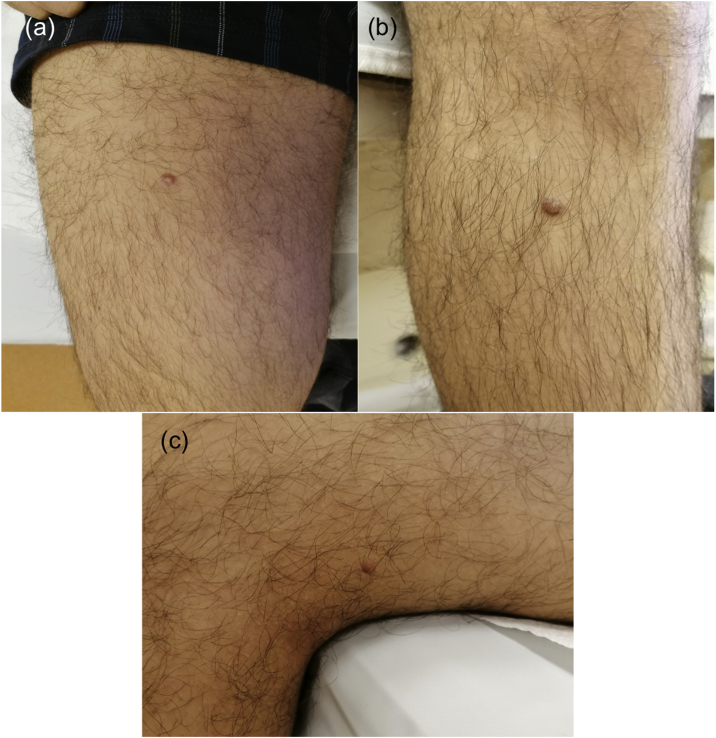
(A–C) Lesions located on lower extremities.

**Figure 2:**
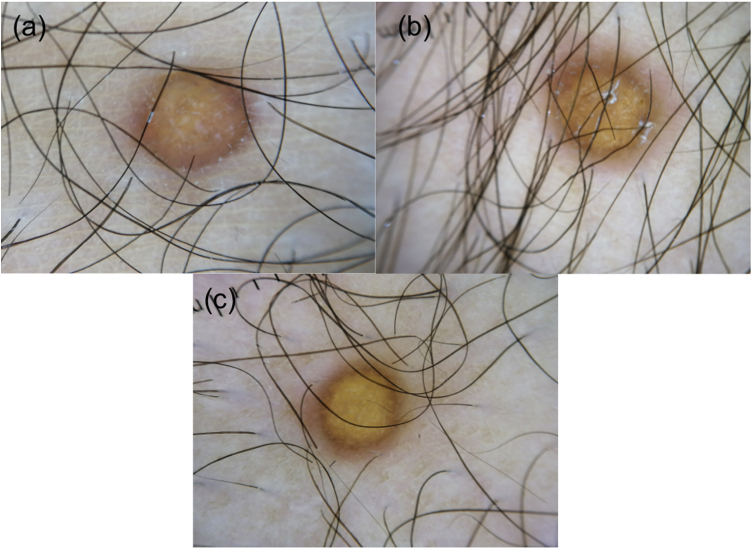
(A–C) Dermoscopy reveals yellow homogenous areas, central white network and pinkish halo.

**Figure 3:**
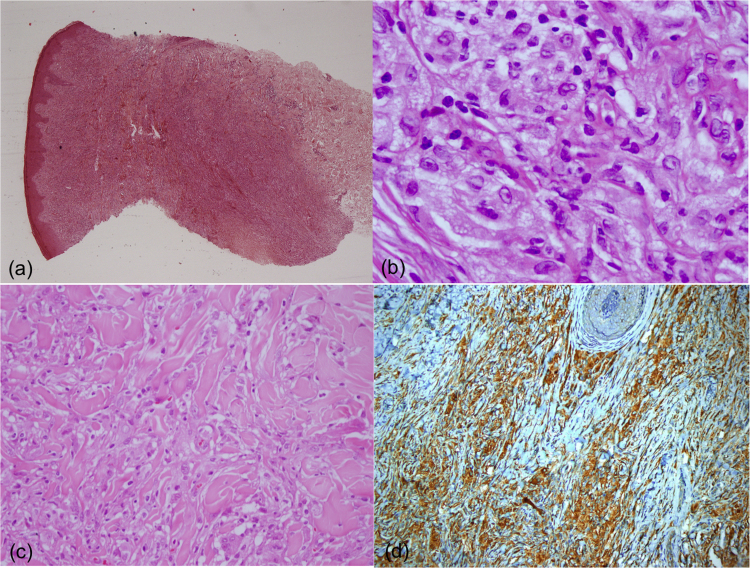
(A) A hypercellular lesion with increased collagen fibers can be seen extending from the superficial dermis to the deep dermis (Hematoxylin & eosin, ×5). (B) Histiocytic cells with vacuolar cytoplasm are noted among dense collagen fibers (Hematoxylin & eosin, ×400). (C) Collagen entrapment at the periphery of the lesion can be seen (Hematoxylin & eosin, ×200). (D) The cells with vacuolar cytoplasm are showing CD68 expression (×20).

## Discussion

Dermatofibromas are common fibrohistiocytic tumors that are mostly diagnosed clinically. However, variants of dermatofibroma sometimes present significant clinical and dermoscopic challenges, and they are best diagnosed by histologic examination.^1^, ^2^

Dermatofibroma is a very common fibrosing cutaneous soft-tissue tumor, typically diagnosed in young to middle-aged adults. Most patients present with a firm, solitary 0.5–1 cm papule, nodule, or plaque, usually brown in color, on the lower extremities.^1^

Lipidized dermatofibroma is a poorly recognized variant of dermatofibroma. It was first reported as lipidized or “ankle-type” fibrous histiocytoma by Calonje and Fletcher in 1994.^3^ Later, two case series investigated the clinical features of lipidized dermatofibroma.^4^, ^5^ Lipidized dermatofibroma represents 2% of dermatofibromas. It usually manifests as a solitary exophytic yellowish papule or nodule.^4^ Compared to ordinary dermatofibromas, lipidized dermatofibroma tends to present with larger solitary lesions and at an older age, mostly in the fifth or sixth decades of life.^4^, ^5^ To our knowledge, there is only one patient presenting with two lesions and one patient under the age of thirty.^5^ Unlike atypical, cellular, and aneurysmal subtypes; this variant of dermatofibroma seems to have a very good prognosis.^1^, ^5^

A recent study evaluating 13 cases revealed three dermoscopic patterns of lipidized dermatofibroma. In the total yellowish homogenous area pattern, the yellowish area involves the whole lesion. The atypical pattern is associated with irregular or centrally located yellowish homogenous areas. The third pattern contains the combination of a central white network and a peripheral delicate pigment network.^4^ In our case, total yellowish homogenous area along with other characteristic features of dermatofibromas such as a central white network and peripheral reddish halo point to the diagnosis of lipidized dermatofibroma. Yellow homogenous area and white network correspond to histiocytes with foamy cytoplasm and to collagenized stroma respectively.^4^

The main differential diagnosis of our patient was Juvenile Xanthogranuloma (JXG). JXG is the most common non-Langerhans cell histiocytosis and typically presents as a solitary well-demarcated, dome-shaped yellowish papule or nodule, mostly on the head and neck of young children.^6^, ^7^ Lesions in children show spontaneous regression within 2-years of diagnosis.^6^ JXG may rarely occur in adults as persistent lesions, mostly in the second to fourth decades. However, similar to lipidized dermatofibroma, JXG may affect older patients.^8^ In contrast to lipidized dermatofibroma which has a predilection for lower extremities, adult JXG tends to involve the head and neck region. While the yellowish papulonodules of JXG typically measure several millimeters in diameter, typical lesions of lipidized dermatofibroma are larger, with a median diameter of 2.5 cm.^8^ “Setting sun” appearance is a typical dermoscopic aspect of JXG with central yellow core and peripheral erythema.^9^ Histopathologically, JXG is characterized by the presence of histiocytes, foam cells, and Touton giant cells. Although typical for JXG, the latter can also be seen in lipidized dermatofibroma. Histiocytes in JXG have more eosinophilic and less lipidized cytoplasm as compared to lipidized dermatofibroma. Observation of an epidermal collarette and a more prominent inflammatory infiltrate that frequently includes eosinophils help distinguish JXG from lipidized dermatofibroma. Furthermore, lipidized dermatofibroma displays a prominent spindle cell component arranged in a storiform pattern and these cells entrap the dermal collagen fibers at the periphery of the lesion. Stromal “wiry” hyalinization, which can sometimes be very extensive, is also a frequent histopathologic feature of lipidized dermatofibroma, differentiating it from JXG.^5^, ^10^ Lastly, several immunohistochemical differences may have a role in the differential diagnosis, such as the presence of CD4 expression in JXG that is not seen in lipidized dermatofibroma.^8^ The disease generally follows a benign course. However, patients with JXG should undergo a complete physical examination regularly. In case of multiple lesions, an ophthalmologic examination should also be performed.^6^

Lipidized dermatofibroma rarely presents in young adults as small papular lesions. This uncommon clinical presentation of lipidized dermatofibromas could be easily mistaken for juvenile xanthogranuloma, cutaneous mastocytoma, or eruptive xanthomata. However, typical dermoscopic and histopathological findings point to the diagnosis of lipidized dermatofibroma, and no further investigations are performed. Lipidized dermatofibromas should be kept in mind in the differential diagnosis of lesions representing yellow areas in dermoscopy.

## Financial support

None declared.

## Authors’ contributions

Tugba KevserUzuncakmak - Participated in data collection; analysis and interpretation and critical literature review; preparation and writing of the manuscript. Muazzez CigdemOba - Participated in data collection; analysis and interpretation and critical literature review; preparation and writing of the manuscript. MehmetSar - Preparation and writing of the manuscript. ZekayiKutlubay - Participated in data collection; analysis and interpretation and critical literature review.

## Conflicts of interest

None declared.
